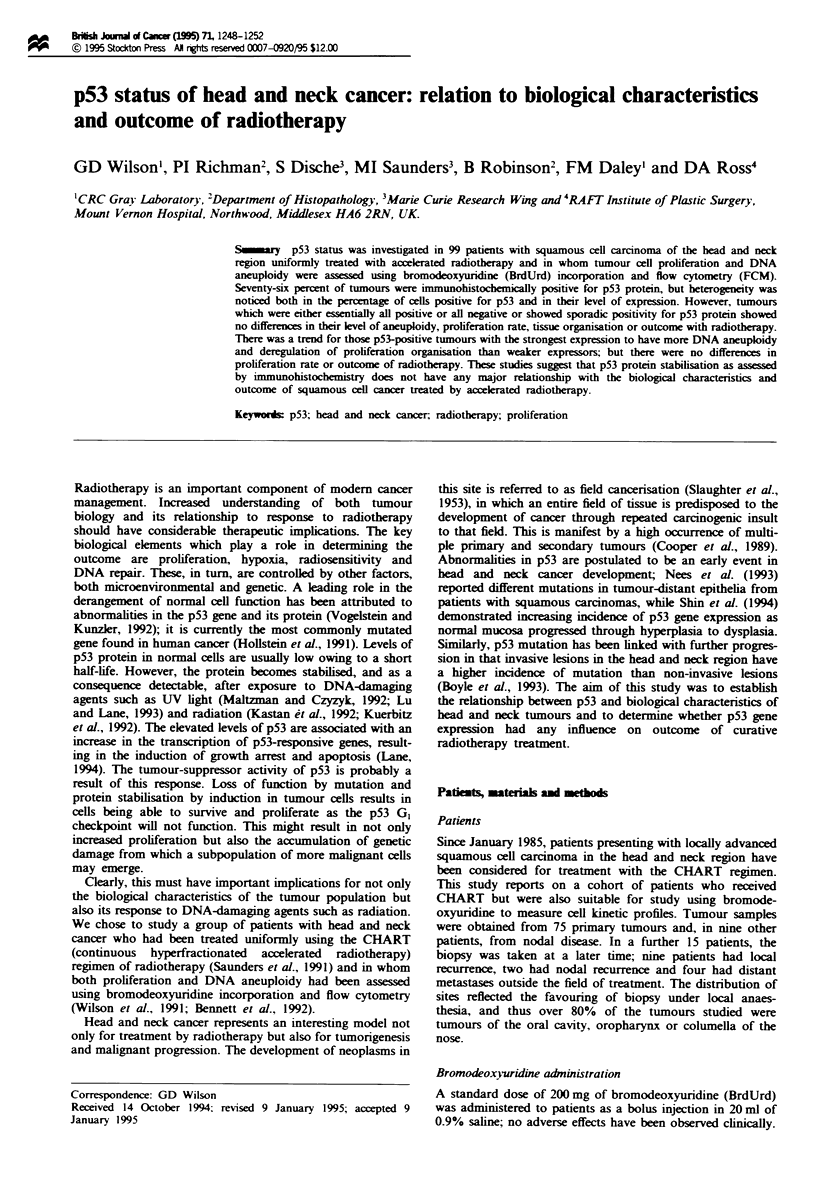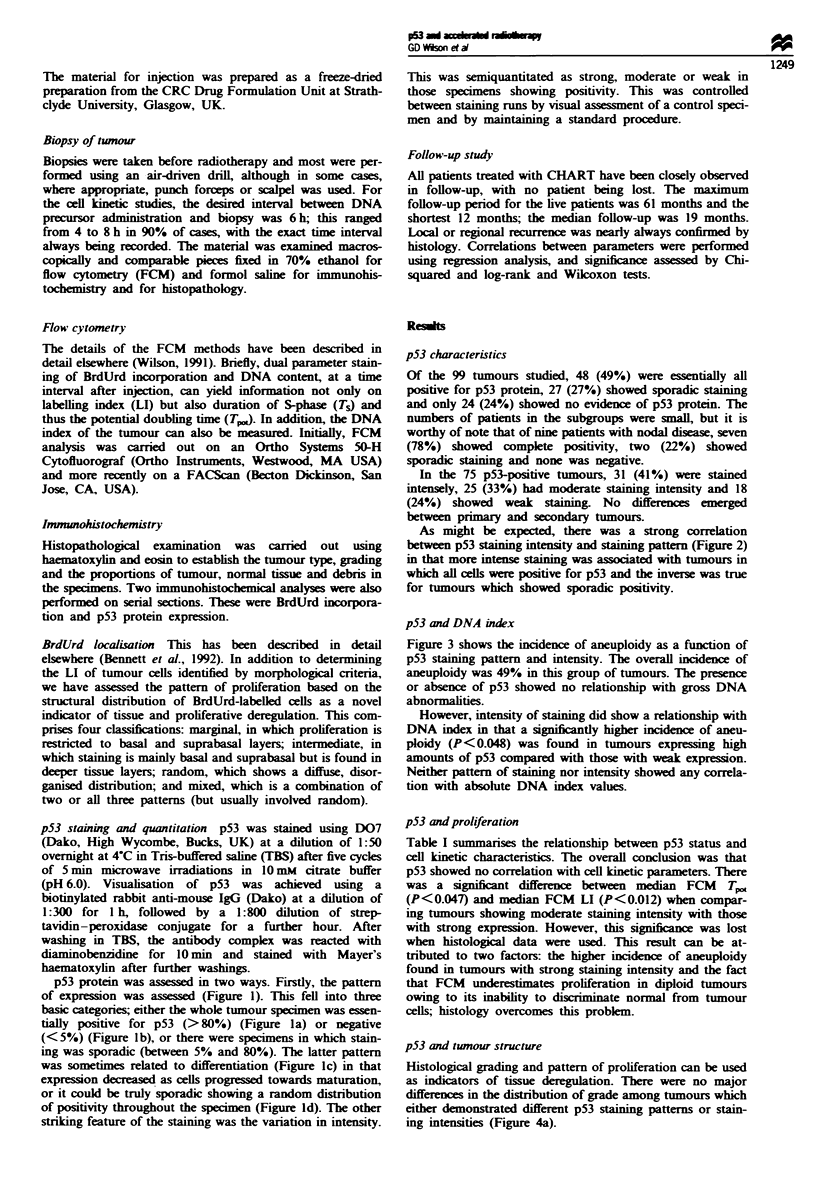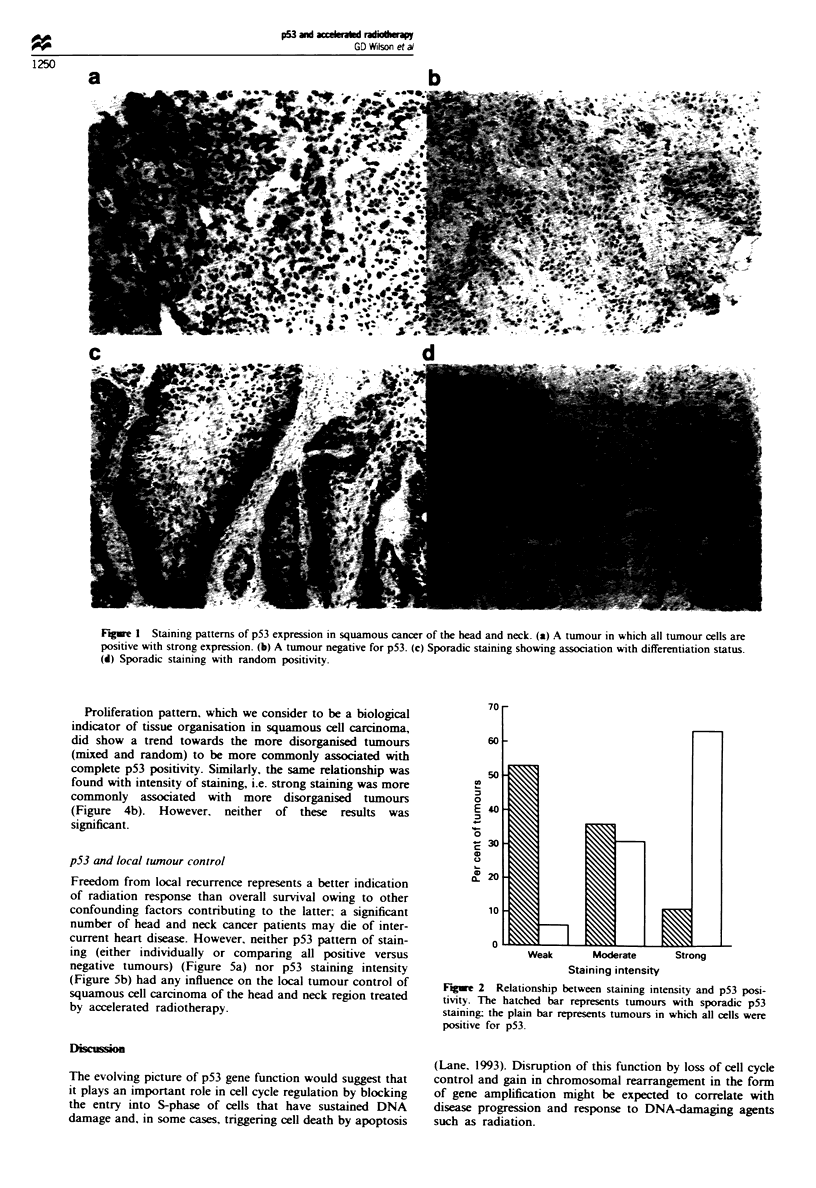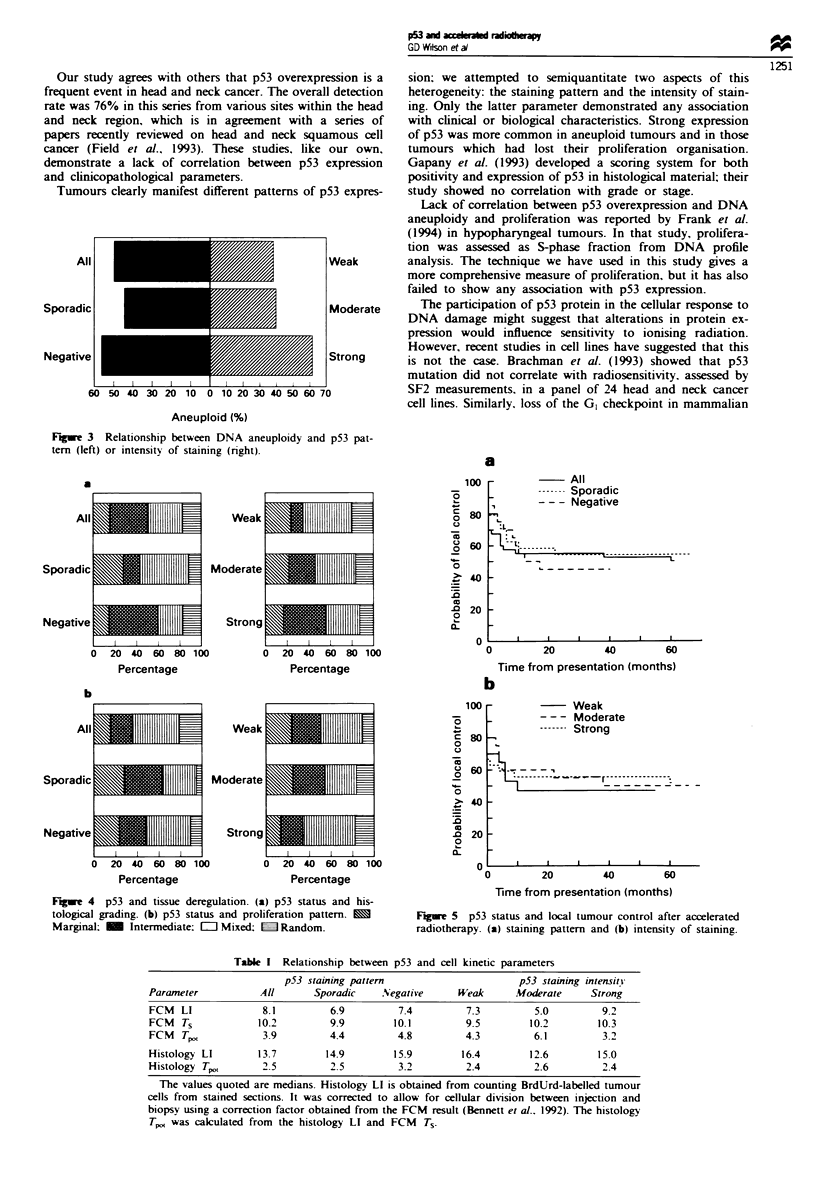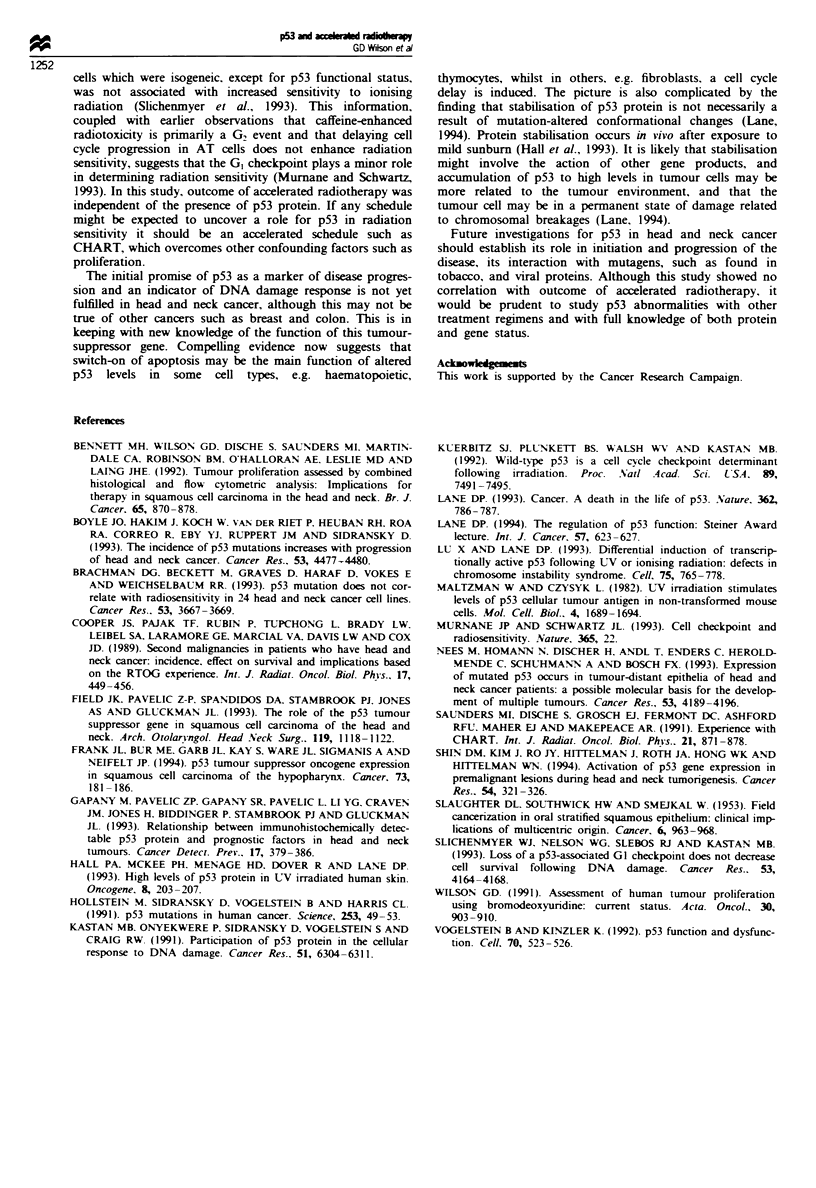# p53 status of head and neck cancer: relation to biological characteristics and outcome of radiotherapy.

**DOI:** 10.1038/bjc.1995.241

**Published:** 1995-06

**Authors:** G. D. Wilson, P. I. Richman, S. Dische, M. I. Saunders, B. Robinson, F. M. Daley, D. A. Ross

**Affiliations:** CRC Gray Laboratory, Mount Vernon Hospital, Northwood, Middlesex, UK.

## Abstract

**Images:**


### p53 status of head and neck cancer: relation to biological characteristics and outcome of radiotherapy

GD Wilson[1], PI Richman[2], S Dische[3], MI Saunders[3], B Robinson[2], FM Daley[1] and DA Ross[4]

[1]*CRC Gray Laboratory,* [2]*Department of Histopathology,* [3]*Marie Curie Research Wing and* [4]*RAFT Institute of Plastic Surgery, Mount Vernon Hospital, Northwood, Middlesex HA6 2RN, UK.*

**Summary** p53 status was investigated in 99 patients with squamous cell carcinoma of the head and neck region uniformly treated with accelerated radiotherapy and in whom tumour cell proliferation and DNA aneuploidy were assessed using bromodeoxyuridine (BrdUrd) incorporation and flow cytometry (FCM). Seventy-six percent of tumours were immunohistochemically positive for p53 protein, but heterogeneity was noticed both in the percentage of cells positive for p53 and in their level of expression. However, tumours which were either essentially all positive or all negative or showed sporadic positivity for p53 protein showed no differences in their level of aneuploidy, proliferation rate, tissue organisation or outcome with radiotherapy. There was a trend for those p53-positive tumours with the strongest expression to have more DNA aneuploidy and deregulation of proliferation organisation than weaker expressors; but there were no differences in proliferation rate or outcome of radiotherapy. These studies suggest that p53 protein stabilisation as assessed by immunohistochemistry does not have any major relationship with the biological characteristics and outcome of squamous cell cancer treated by accelerated radiotherapy.

**Keywords:** p53; head and neck cancer; radiotherapy; proliferation

Radiotherapy is an important component of modern cancer management. Increased understanding of both tumour biology and its relationship to response to radiotherapy should have considerable therapeutic implications. The key biological elements which play a role in determining the outcome are proliferation, hypoxia, radiosensitivity and DNA repair. These, in turn, are controlled by other factors, both microenvironmental and genetic. A leading role in the derangement of normal cell function has been attributed to abnormalities in the p53 gene and its protein (Vogelstein and Kunzler, 1992); it is currently the most commonly mutated gene found in human cancer (Hollstein *et al.*, 1991). Levels of p53 protein in normal cells are usually low owing to a short half-life. However, the protein becomes stabilised, and as a consequence detectable, after exposure to DNA-damaging agents such as UV light (Maltzman and Czyzyk, 1992; Lu and Lane, 1993) and radiation (Kastan *et al.*, 1992; Kuerbitz *et al.*, 1992). The elevated levels of p53 are associated with an increase in the transcription of p53-responsive genes, resulting in the induction of growth arrest and apoptosis (Lane, 1994). The tumour-suppressor activity of p53 is probably a result of this response. Loss of function by mutation and protein stabilisation by induction in tumour cells results in cells being able to survive and proliferate as the p53 $G_1$ checkpoint will not function. This might result in not only increased proliferation but also the accumulation of genetic damage from which a subpopulation of more malignant cells may emerge.

Clearly, this must have important implications for not only the biological characteristics of the tumour population but also its response to DNA-damaging agents such as radiation. We chose to study a group of patients with head and neck cancer who had been treated uniformly using the CHART (continuous hyperfractionated accelerated radiotherapy) regimen of radiotherapy (Saunders *et al.*, 1991) and in whom both proliferation and DNA aneuploidy had been assessed using bromodeoxyuridine incorporation and flow cytometry (Wilson *et al.*, 1991; Bennett *et al.*, 1992).

Head and neck cancer represents an interesting model not only for treatment by radiotherapy but also for tumorigenesis and malignant progression. The development of neoplasms in this site is referred to as field cancerisation (Slaughter *et al.*, 1953), in which an entire field of tissue is predisposed to the development of cancer through repeated carcinogenic insult to that field. This is manifest by a high occurrence of multiple primary and secondary tumours (Cooper *et al.*, 1989). Abnormalities in p53 are postulated to be an early event in head and neck cancer development; Nees *et al.* (1993) reported different mutations in tumour-distant epithelia from patients with squamous carcinomas, while Shin *et al.* (1994) demonstrated increasing incidence of p53 gene expression as normal mucosa progressed through hyperplasia to dysplasia. Similarly, p53 mutation has been linked with further progression in that invasive lesions in the head and neck region have a higher incidence of mutation than non-invasive lesions (Boyle *et al.*, 1993). The aim of this study was to establish the relationship between p53 and biological characteristics of head and neck tumours and to determine whether p53 gene expression had any influence on outcome of curative radiotherapy treatment.

### Patients, materials and methods

### *Patients*

Since January 1985, patients presenting with locally advanced squamous cell carcinoma in the head and neck region have been considered for treatment with the CHART regimen. This study reports on a cohort of patients who received CHART but were also suitable for study using bromodeoxyuridine to measure cell kinetic profiles. Tumour samples were obtained from 75 primary tumours and, in nine other patients, from nodal disease. In a further 15 patients, the biopsy was taken at a later time; nine patients had local recurrence, two had nodal recurrence and four had distant metastases outside the field of treatment. The distribution of sites reflected the favouring of biopsy under local anaesthesia, and thus over 80% of the tumours studied were tumours of the oral cavity, oropharynx or columella of the nose.

### *Bromodeoxyuridine administration*

A standard dose of 200 mg of bromodeoxyuridine (BrdUrd) was administered to patients as a bolus injection in 20 ml of 0.9% saline; no adverse effects have been observed clinically.

Correspondence: GD Wilson
Received 14 October 1994; revised 9 January 1995; accepted 9 January 1995

The material for injection was prepared as a freeze-dried preparation from the CRC Drug Formulation Unit at Strathclyde University, Glasgow, UK.

*Biopsy of tumour*

Biopsies were taken before radiotherapy and most were performed using an air-driven drill, although in some cases, where appropriate, punch forceps or scalpel was used. For the cell kinetic studies, the desired interval between DNA precursor administration and biopsy was 6 h; this ranged from 4 to 8 h in 90% of cases, with the exact time interval always being recorded. The material was examined macroscopically and comparable pieces fixed in 70% ethanol for flow cytometry (FCM) and formol saline for immunohistochemistry and for histopathology.

*Flow cytometry*

The details of the FCM methods have been described in detail elsewhere (Wilson, 1991). Briefly, dual parameter staining of BrdUrd incorporation and DNA content, at a time interval after injection, can yield information not only on labelling index (LI) but also duration of S-phase ($T_S$) and thus the potential doubling time ($T_{pot}$). In addition, the DNA index of the tumour can also be measured. Initially, FCM analysis was carried out on an Ortho Systems 50-H Cytofluorograf (Ortho Instruments, Westwood, MA USA) and more recently on a FACScan (Becton Dickinson, San Jose, CA, USA).

*Immunohistochemistry*

Histopathological examination was carried out using haematoxylin and eosin to establish the tumour type, grading and the proportions of tumour, normal tissue and debris in the specimens. Two immunohistochemical analyses were also performed on serial sections. These were BrdUrd incorporation and p53 protein expression.

*BrdUrd localisation* This has been described in detail elsewhere (Bennett *et al.*, 1992). In addition to determining the LI of tumour cells identified by morphological criteria, we have assessed the pattern of proliferation based on the structural distribution of BrdUrd-labelled cells as a novel indicator of tissue and proliferative deregulation. This comprises four classifications: marginal, in which proliferation is restricted to basal and suprabasal layers; intermediate, in which staining is mainly basal and suprabasal but is found in deeper tissue layers; random, which shows a diffuse, disorganised distribution; and mixed, which is a combination of two or all three patterns (but usually involved random).

*p53 staining and quantitation* p53 was stained using DO7 (Dako, High Wycombe, Bucks, UK) at a dilution of 1:50 overnight at 4°C in Tris-buffered saline (TBS) after five cycles of 5 min microwave irradiations in 10 mM citrate buffer (pH 6.0). Visualisation of p53 was achieved using a biotinylated rabbit anti-mouse IgG (Dako) at a dilution of 1:300 for 1 h, followed by a 1:800 dilution of streptavidin-peroxidase conjugate for a further hour. After washing in TBS, the antibody complex was reacted with diaminobenzidine for 10 min and stained with Mayer's haematoxylin after further washings.

p53 protein was assessed in two ways. Firstly, the pattern of expression was assessed (Figure 1). This fell into three basic categories; either the whole tumour specimen was essentially positive for p53 (>80%) (Figure 1a) or negative (<5%) (Figure 1b), or there were specimens in which staining was sporadic (between 5% and 80%). The latter pattern was sometimes related to differentiation (Figure 1c) in that expression decreased as cells progressed towards maturation, or it could be truly sporadic showing a random distribution of positivity throughout the specimen (Figure 1d). The other striking feature of the staining was the variation in intensity. This was semiquantitated as strong, moderate or weak in those specimens showing positivity. This was controlled between staining runs by visual assessment of a control specimen and by maintaining a standard procedure.

*Follow-up study*

All patients treated with CHART have been closely observed in follow-up, with no patient being lost. The maximum follow-up period for the live patients was 61 months and the shortest 12 months; the median follow-up was 19 months. Local or regional recurrence was nearly always confirmed by histology. Correlations between parameters were performed using regression analysis, and significance assessed by Chi-squared and log-rank and Wilcoxon tests.

### Results

*p53 characteristics*

Of the 99 tumours studied, 48 (49%) were essentially all positive for p53 protein, 27 (27%) showed sporadic staining and only 24 (24%) showed no evidence of p53 protein. The numbers of patients in the subgroups were small, but it is worthy of note that of nine patients with nodal disease, seven (78%) showed complete positivity, two (22%) showed sporadic staining and none was negative.

In the 75 p53-positive tumours, 31 (41%) were stained intensely, 25 (33%) had moderate staining intensity and 18 (24%) showed weak staining. No differences emerged between primary and secondary tumours.

As might be expected, there was a strong correlation between p53 staining intensity and staining pattern (Figure 2) in that more intense staining was associated with tumours in which all cells were positive for p53 and the inverse was true for tumours which showed sporadic positivity.

*p53 and DNA index*

Figure 3 shows the incidence of aneuploidy as a function of p53 staining pattern and intensity. The overall incidence of aneuploidy was 49% in this group of tumours. The presence or absence of p53 showed no relationship with gross DNA abnormalities.

However, intensity of staining did show a relationship with DNA index in that a significantly higher incidence of aneuploidy ($P<0.048$) was found in tumours expressing high amounts of p53 compared with those with weak expression. Neither pattern of staining nor intensity showed any correlation with absolute DNA index values.

*p53 and proliferation*

Table I summarises the relationship between p53 status and cell kinetic characteristics. The overall conclusion was that p53 showed no correlation with cell kinetic parameters. There was a significant difference between median FCM $T_{pot}$ ($P<0.047$) and median FCM LI ($P<0.012$) when comparing tumours showing moderate staining intensity with those with strong expression. However, this significance was lost when histological data were used. This result can be attributed to two factors: the higher incidence of aneuploidy found in tumours with strong staining intensity and the fact that FCM underestimates proliferation in diploid tumours owing to its inability to discriminate normal from tumour cells; histology overcomes this problem.

*p53 and tumour structure*

Histological grading and pattern of proliferation can be used as indicators of tissue deregulation. There were no major differences in the distribution of grade among tumours which either demonstrated different p53 staining patterns or staining intensities (Figure 4a).

**Figure 1** Staining patterns of p53 expression in squamous cancer of the head and neck. (**a**) A tumour in which all tumour cells are positive with strong expression. (**b**) A tumour negative for p53. (**c**) Sporadic staining showing association with differentiation status. (**d**) Sporadic staining with random positivity.

Proliferation pattern, which we consider to be a biological indicator of tissue organisation in squamous cell carcinoma, did show a trend towards the more disorganised tumours (mixed and random) to be more commonly associated with complete p53 positivity. Similarly, the same relationship was found with intensity of staining, i.e. strong staining was more commonly associated with more disorganised tumours (Figure 4b). However, neither of these results was significant.

*p53 and local tumour control*

Freedom from local recurrence represents a better indication of radiation response than overall survival owing to other confounding factors contributing to the latter; a significant number of head and neck cancer patients may die of intercurrent heart disease. However, neither p53 pattern of staining (either individually or comparing all positive versus negative tumours) (Figure 5a) nor p53 staining intensity (Figure 5b) had any influence on the local tumour control of squamous cell carcinoma of the head and neck region treated by accelerated radiotherapy.

**Figure 2** Relationship between staining intensity and p53 positivity. The hatched bar represents tumours with sporadic p53 staining; the plain bar represents tumours in which all cells were positive for p53.

### Discussion

The evolving picture of p53 gene function would suggest that it plays an important role in cell cycle regulation by blocking the entry into S-phase of cells that have sustained DNA damage and, in some cases, triggering cell death by apoptosis (Lane, 1993). Disruption of this function by loss of cell cycle control and gain in chromosomal rearrangement in the form of gene amplification might be expected to correlate with disease progression and response to DNA-damaging agents such as radiation.

Our study agrees with others that p53 overexpression is a frequent event in head and neck cancer. The overall detection rate was 76% in this series from various sites within the head and neck region, which is in agreement with a series of papers recently reviewed on head and neck squamous cell cancer (Field *et al.*, 1993). These studies, like our own, demonstrate a lack of correlation between p53 expression and clinicopathological parameters.

Tumours clearly manifest different patterns of p53 expression; we attempted to semiquantitate two aspects of this heterogeneity: the staining pattern and the intensity of staining. Only the latter parameter demonstrated any association with clinical or biological characteristics. Strong expression of p53 was more common in aneuploid tumours and in those tumours which had lost their proliferation organisation. Gapany *et al.* (1993) developed a scoring system for both positivity and expression of p53 in histological material; their study showed no correlation with grade or stage.

Lack of correlation between p53 overexpression and DNA aneuploidy and proliferation was reported by Frank *et al.* (1994) in hypopharyngeal tumours. In that study, proliferation was assessed as S-phase fraction from DNA profile analysis. The technique we have used in this study gives a more comprehensive measure of proliferation, but it has also failed to show any association with p53 expression.

The participation of p53 protein in the cellular response to DNA damage might suggest that alterations in protein expression would influence sensitivity to ionising radiation. However, recent studies in cell lines have suggested that this is not the case. Brachman *et al.* (1993) showed that p53 mutation did not correlate with radiosensitivity, assessed by SF2 measurements, in a panel of 24 head and neck cancer cell lines. Similarly, loss of the $G_1$ checkpoint in mammalian

**Figure 3** Relationship between DNA aneuploidy and p53 pattern (left) or intensity of staining (right).

**Figure 4** p53 and tissue deregulation. (**a**) p53 status and histological grading. (**b**) p53 status and proliferation pattern. ▧ Marginal; ■ Intermediate; □ Mixed; ▤ Random.

**Figure 5** p53 status and local tumour control after accelerated radiotherapy. (**a**) staining pattern and (**b**) intensity of staining.

**Table I** Relationship between p53 and cell kinetic parameters

| | p53 staining pattern | | | p53 staining intensity | | |
|---|---|---|---|---|---|---|
| *Parameter* | *All* | *Sporadic* | *Negative* | *Weak* | *Moderate* | *Strong* |
| FCM LI | 8.1 | 6.9 | 7.4 | 7.3 | 5.0 | 9.2 |
| FCM $T_S$ | 10.2 | 9.9 | 10.1 | 9.5 | 10.2 | 10.3 |
| FCM $T_{pot}$ | 3.9 | 4.4 | 4.8 | 4.3 | 6.1 | 3.2 |
| Histology LI | 13.7 | 14.9 | 15.9 | 16.4 | 12.6 | 15.0 |
| Histology $T_{pot}$ | 2.5 | 2.5 | 3.2 | 2.4 | 2.6 | 2.4 |

The values quoted are medians. Histology LI is obtained from counting BrdUrd-labelled tumour cells from stained sections. It was corrected to allow for cellular division between injection and biopsy using a correction factor obtained from the FCM result (Bennett *et al.*, 1992). The histology $T_{pot}$ was calculated from the histology LI and FCM $T_S$.

cells which were isogeneic, except for p53 functional status, was not associated with increased sensitivity to ionising radiation (Slichenmyer et al., 1993). This information, coupled with earlier observations that caffeine-enhanced radiotoxicity is primarily a $G_2$ event and that delaying cell cycle progression in AT cells does not enhance radiation sensitivity, suggests that the $G_1$ checkpoint plays a minor role in determining radiation sensitivity (Murnane and Schwartz, 1993). In this study, outcome of accelerated radiotherapy was independent of the presence of p53 protein. If any schedule might be expected to uncover a role for p53 in radiation sensitivity it should be an accelerated schedule such as CHART, which overcomes other confounding factors such as proliferation.

The initial promise of p53 as a marker of disease progression and an indicator of DNA damage response is not yet fulfilled in head and neck cancer, although this may not be true of other cancers such as breast and colon. This is in keeping with new knowledge of the function of this tumour-suppressor gene. Compelling evidence now suggests that switch-on of apoptosis may be the main function of altered p53 levels in some cell types, e.g. haematopoietic, thymocytes, whilst in others, e.g. fibroblasts, a cell cycle delay is induced. The picture is also complicated by the finding that stabilisation of p53 protein is not necessarily a result of mutation-altered conformational changes (Lane, 1994). Protein stabilisation occurs *in vivo* after exposure to mild sunburn (Hall et al., 1993). It is likely that stabilisation might involve the action of other gene products, and accumulation of p53 to high levels in tumour cells may be more related to the tumour environment, and that the tumour cell may be in a permanent state of damage related to chromosomal breakages (Lane, 1994).

Future investigations for p53 in head and neck cancer should establish its role in initiation and progression of the disease, its interaction with mutagens, such as found in tobacco, and viral proteins. Although this study showed no correlation with outcome of accelerated radiotherapy, it would be prudent to study p53 abnormalities with other treatment regimens and with full knowledge of both protein and gene status.

**Acknowledgements**

This work is supported by the Cancer Research Campaign.

**References**

BENNETT MH, WILSON GD, DISCHE S, SAUNDERS MI, MARTINDALE CA, ROBINSON BM, O'HALLORAN AE, LESLIE MD AND LAING JHE. (1992). Tumour proliferation assessed by combined histological and flow cytometric analysis: Implications for therapy in squamous cell carcinoma in the head and neck. *Br. J. Cancer*, **65**, 870-878.

BOYLE JO, HAKIM J, KOCH W, VAN DER RIET P, HEUBAN RH, ROA RA, CORREO R, EBY YJ, RUPPERT JM AND SIDRANSKY D. (1993). The incidence of p53 mutations increases with progression of head and neck cancer. *Cancer Res.*, **53**, 4477-4480.

BRACHMAN DG, BECKETT M, GRAVES D, HARAF D, VOKES E AND WEICHSELBAUM RR. (1993). p53 mutation does not correlate with radiosensitivity in 24 head and neck cancer cell lines. *Cancer Res.*, **53**, 3667-3669.

COOPER JS, PAJAK TF, RUBIN P, TUPCHONG L, BRADY LW, LEIBEL SA, LARAMORE GE, MARCIAL VA, DAVIS LW AND COX JD. (1989). Second malignancies in patients who have head and neck cancer: incidence, effect on survival and implications based on the RTOG experience. *Int. J. Radiat. Oncol. Biol. Phys.*, **17**, 449-456.

FIELD JK, PAVELIC Z-P, SPANDIDOS DA, STAMBROOK PJ, JONES AS AND GLUCKMAN JL. (1993). The role of the p53 tumour suppressor gene in squamous cell carcinoma of the head and neck. *Arch. Otolaryngol. Head Neck Surg.*, **119**, 1118-1122.

FRANK JL, BUR ME, GARB JL, KAY S, WARE JL, SIGMANIS A AND NEIFELT JP. (1994). p53 tumour suppressor oncogene expression in squamous cell carcinoma of the hypopharynx. *Cancer*, **73**, 181-186.

GAPANY M, PAVELIC ZP, GAPANY SR, PAVELIC L, LI YG, CRAVEN JM, JONES H, BIDDINGER P, STAMBROOK PJ AND GLUCKMAN JL. (1993). Relationship between immunohistochemically detectable p53 protein and prognostic factors in head and neck tumours. *Cancer Detect. Prev.*, **17**, 379-386.

HALL PA, MCKEE PH, MENAGE HD, DOVER R AND LANE DP. (1993). High levels of p53 protein in UV irradiated human skin. *Oncogene*, **8**, 203-207.

HOLLSTEIN M, SIDRANSKY D, VOGELSTEIN B AND HARRIS CL. (1991). p53 mutations in human cancer. *Science*, **253**, 49-53.

KASTAN MB, ONYEKWERE P, SIDRANSKY D, VOGELSTEIN S AND CRAIG RW. (1991). Participation of p53 protein in the cellular response to DNA damage. *Cancer Res.*, **51**, 6304-6311.

KUERBITZ SJ, PLUNKETT BS, WALSH WV AND KASTAN MB. (1992). Wild-type p53 is a cell cycle checkpoint determinant following irradiation. *Proc. Natl Acad. Sci. USA*, **89**, 7491-7495.

LANE DP. (1993). Cancer. A death in the life of p53. *Nature*, **362**, 786-787.

LANE DP. (1994). The regulation of p53 function: Steiner Award lecture. *Int. J. Cancer*, **57**, 623-627.

LU X AND LANE DP. (1993). Differential induction of transcriptionally active p53 following UV or ionising radiation: defects in chromosome instability syndrome. *Cell*, **75**, 765-778.

MALTZMAN W AND CZYSYK L. (1982). UV irradiation stimulates levels of p53 cellular tumour antigen in non-transformed mouse cells. *Mol. Cell. Biol.*, **4**, 1689-1694.

MURNANE JP AND SCHWARTZ JL. (1993). Cell checkpoint and radiosensitivity. *Nature*, **365**, 22.

NEES M, HOMANN N, DISCHER H, ANDL T, ENDERS C, HEROLD-MENDE C, SCHUHMANN A AND BOSCH FX. (1993). Expression of mutated p53 occurs in tumour-distant epithelia of head and neck cancer patients: a possible molecular basis for the development of multiple tumours. *Cancer Res.*, **53**, 4189-4196.

SAUNDERS MI, DISCHE S, GROSCH EJ, FERMONT DC, ASHFORD RFU, MAHER EJ AND MAKEPEACE AR. (1991). Experience with CHART. *Int. J. Radiat. Oncol. Biol. Phys.*, **21**, 871-878.

SHIN DM, KIM J, RO JY, HITTELMAN J, ROTH JA, HONG WK AND HITTELMAN WN. (1994). Activation of p53 gene expression in premalignant lesions during head and neck tumorigenesis. *Cancer Res.*, **54**, 321-326.

SLAUGHTER DL, SOUTHWICK HW AND SMEJKAL W. (1953). Field cancerization in oral stratified squamous epithelium: clinical implications of multicentric origin. *Cancer*, **6**, 963-968.

SLICHENMYER WJ, NELSON WG, SLEBOS RJ AND KASTAN MB. (1993). Loss of a p53-associated G1 checkpoint does not decrease cell survival following DNA damage. *Cancer Res.*, **53**, 4164-4168.

WILSON GD. (1991). Assessment of human tumour proliferation using bromodeoxyuridine: current status. *Acta. Oncol.*, **30**, 903-910.

VOGELSTEIN B AND KINZLER K. (1992). p53 function and dysfunction. *Cell*, **70**, 523-526.